# Variant rs9939609 in the *FTO *gene is associated with body mass index among Chinese children

**DOI:** 10.1186/1471-2350-11-136

**Published:** 2010-09-22

**Authors:** Hongyun Fang, Yanping Li, Songming Du, Xiaoqi Hu, Qian Zhang, Ailing Liu, Guansheng Ma

**Affiliations:** 1 National Institute for Nutrition and Food Safety, Chinese Center for Disease Control and Prevention, Beijing, China

## Abstract

**Background:**

Fat-mass and obesity-associated (*FTO*) gene is a gene located in chromosome region 16q12.2. Genetic variants in *FTO *are associated with the obesity phenotype in European and Hispanic populations. However, this association still remains controversial in Asian population. We aimed to test the association of *FTO *genetic variants with obesity and obesity-related metabolic traits among children living in Beijing, China.

**Methods:**

We genotyped *FTO *variants rs9939609 in 670 children (332 girls and 338 boys) aged 8-11 years living in Beijing, and analyzed its association with obesity and obesity-related metabolic traits. Overweight and obesity were defined by age- and sex-specific BMI reference for Chinese children. Obesity-related metabolic traits included fasting plasma glucose, lipid profiles, leptin, ghrelin, adiponectin and blood pressures.

**Results:**

The frequency of rs9939609 A allele was 12.2%, which was 21.9% for the heterozygote and 1.2% for the homozygote of the A allele. The obesity prevalence among the carriers of AA/AT genotypes was significantly higher than that among those with TT genotype (36.4% *vs*. 22.6%, *P *= 0.004). Compared to the carrier of TT genotype, the likelihood of obesity was 1.79 (95% confidence interval (95% CI) 1.20-2.67, *P *= 0.004) for the carrier of AA/AT genotype, after adjustment of sex, age and puberty stages. The BMI Z-score of children with AA/AT genotype were significantly higher than that of their counterparts with the TT genotype (1.1 ± 0.1 *vs*. 0.8 ± 0.1, *P *= 0.02). The concentration of triglyceride was 1.03 ± 0.52 mmol/L among TT carrier and 1.13 ± 0.68 mmol/L among AA/AT carrier (*P *= 0.045). While, the concentrations of adiponectin were 18.0 ± 0.4 μg/ml among carriers of TT and 16.2 ± 0.7 μg/ml among subjects with AA/AT genotype (*P *= 0.03). The level of glucose marginally increased in the AA/AT genotype subjects (4.67 ± 0.40 mmol/L *vs*. 4.60 ± 0.35 mmol/L, *P *= 0.08). The evidence of association was reduced after adjustment for BMI (*P *= 0.38 for triglyceride, *P *= 0.20 for adiponectin and glucose). There was weak evidence of association between rs9939609 and other obesity-related metabolic traits including total cholesterol (3.92 ± 0.03 mmol/L *vs*. 4.02 ± 0.05 mmol/L, *P *= 0.10), insulin (2.69 ± 1.77 ng/ml *vs*. 3.12 ± 2.91 ng/ml, *P *= 0.14), and insulin resistance (HOMA-IR 0.56 ± 0.03 *vs*. 0.66 ± 0.05, *P *= 0.10).

**Conclusions:**

Genetic variation in the *FTO *gene associates with obesity in Chinese children.

## Background

Obesity is a consequence of unhealthy lifestyle combined with genetic susceptibility. Recent genome-wide association (GWA) studies identified a group of loci associated with obesity. Among those genes, *FTO *explained the largest variation of BMI and obesity [1,2]. Frayling et al. first reported the association of *FTO *genetic variants with obesity [1]. Subsequently, the association with BMI and obesity was unequivocally replicated in European and Hispanic populations in both children and adults [3-8].

However, these associations were controversial with regard to Asian populations [9-18]. Chang et al. [9] reported that rs9939609 was associated with BMI in the Chinese population living in Taiwan, China. Tan et al. [10] also found associations of rs9939609 and other SNPs located in the intron 1 LD block with obesity in the Chinese and Malays lived in Singapore. Omori S and Hotta K [11,12] found strong associations of *FTO *variants with BMI in the Japanese population. Cha et al. [13] confirmed the strong association of *FTO *genetic variants with BMI in a Korean population. Ng et al. found association of *FTO *variants with BMI in East Asians but the association of *FTO *and BMI was weaker in Asians as compared to the Europeans [14]. In contrast, the first study of the association between *FTO *and BMI among Chinese population in mainland China [15] found no association of *FTO *variants with obesity and BMI in the Chinese adults aged 50-70 years living in Beijing and Shanghai. Meanwhile, Horikoshi et al. [16] also found no association of *FTO *variants with BMI in the Japanese population. The rs9939609 A allele frequency was lower in Chinese and Japanese compared to that in Europeans, which was assumed to be one reason of the controversial findings. Recently, Li X et al. [17] found that rs9939609 SNP was strongly associated with risk of obesity and newly diagnosed type 2 diabetes in the Chinese adults and Xi et al. [18] confirmed that *FTO *rs9939609 variant was strongly associated with BMI and the risk of obesity in Chinese children and adolescents in Beijing.

The purpose of this study is to replicate the associations of *FTO *rs9939609 with obesity and obesity-related metabolic traits in Chinese children.

## Methods

### Study participants

This is a cross-sectional study conducted in 2005. Two districts, Dongcheng and Chongwen, were randomly selected in downtown Beijing, China. Ten primary schools were randomly selected from the two districts. Han students of the third and fourth grade in the selected schools were asked to voluntarily participate in the study. In this study, we restrict our analysis to Han nationality to minimize the probably effect from population stratification.

A total of 1126 Han students aged 8-11 years and/or their legal guardians were invited and 674 agreed to participate and signed the informed consent form, with a participation rate of 59.9%. All the 674 Han students were enrolled and voluntarily attended a clinical examination that included standardized anthropometric measurements and fasting plasma samples collection. A total of 670 students were successfully isolated genomic DNA and genotyped *FTO *rs9939609 genotypes. No significant differences were found of age, sex, weight, height and BMI between the students who participated and who did not.

The study protocol was approved by the Ethical Committee of the National Institute for Nutrition and Food Safety, Chinese Center for Disease Control and Prevention. All study participants and/or legal guardians provided written informed consent.

### Anthropometric and laboratory methods

Anthropometric measurements included weight, height, waist circumference, hip circumference, subscapular skinfold thickness, suprailiac skinfold thickness, systolic (SBP) and diastolic blood pressures (DBP). All anthropometric measurements were taken in accordance with WHO standards [19] by the trained investigators in the schools. Anthropometric measurements for each participant were taken after an overnight fast while the subject wore light clothing and no shoes.

Height was determined using a standard steel strip stadiometer and was measured to the nearest 0.1 cm. Weight was measured to the nearest 0.1 kg using a digital electronic scale (Seca, model 890, Hamburg). Waist circumference was measured mid-way between the lower rib margin and the iliac crest with flexible anthropometric tape. Hip circumference was measured at the point over the buttocks yielding the maximum circumference. The waist circumference and hip circumference were measured twice to the nearest 0.1 cm. If the variation between these two measurements was greater than 2 cm, a third measurement was taken and the mean was calculated by using the two closest measurements. The BMI Z-score, based on age and sex, was calculated according to the method recommended by WHO [20] using the WHO growth reference for children aged 5-17 years [21].

Skinfolds were lifted vertically and then measuring its thickness with Harpenden Skinfold Caliper. The subscapular skinfold was measured below the tip of the scapula. The suprailiac skinfold was measured immediately above the crest of the ilium, and the fold was lifted at a slight angle to the vertical along the normal fold line. The skinfolds were measured twice to the nearest 0.2 mm. If the variation between these two measurements was greater than 0.5 mm, a third measurement was taken and the mean was calculated by using the two closest measurements.

Blood pressure was measured in the supine position using a mercury sphygmomanometer by trained nurses. Subjects took at least a 10-min rest before the measurement was taken. Three measurements were taken from all the subjects at 2-min intervals, and the average of the last two measurements was used. These were recorded to the nearest 2 mmHg.

Bioelectrical impedance analysis (BIA) method was used to assess the body composition. A four terminal impedance plethysmograph (RJL2System 101 USA, 50 kHz, 800 μA) was used to measure electrical impedance, resistance (R) and reactance (Xc), according to the instructions provided by the manufacturer. Fat free mass (FFM), fat mass and percent body fat were calculated using the prediction equations suggested by Deurenberg *et al. *[22].

Pubertal staging criteria and definitions used were the 5 stages of pubic hair growth, breast development, and genital development described by Marshall and Tanner known as Tanner staging [23,24]. Data were collected by physicians during the physical examination.

Blood samples were taken in the morning after an overnight fasting. Plasma glucose was determined by a glucose-oxidase method (Osaka, Japan). Total cholesterol (TC), triglycerides (TG), low density lipoprotein cholesterol (LDL) and high density lipoprotein cholesterol (HDL) were determined by enzymatic methods using commercial kits (Fuji Film, Tokyo, Japan). The intra and inter coefficient variations (CV) were less than 16% for all these tests. Serum insulin concentrations were determined by radioimmunoassay (RIA) (Human Insulin Specific RIA kit, Linco Research, St Louis, MO, USA). The intra and inter coefficient variations were 7.2% and 15.1%, respectively. Leptin and adiponectin serum concentrations were assayed by sensitive/specific RIA as follows: leptin by a highly sensitive RIA (Human Leptin RIA Kit, Linco Research, St Louis, MO, USA), the intra and inter coefficient variations were 9.3% and 14.5%, respectively; Total adiponectin were determined by competitive radioimunoassay (Human Adiponectin Specific RIA kit, Linco Research, St Louis, MO, USA), the intra and inter coefficient variations were 7.5% and 15.8%, respectively. Total ghrelin concentrations were determined by radioimmunoassay (RIA) kit (Human Ghrelin (Total) RIA Kit, Linco Research, St. Charles, MO, USA), and the intra and inter coefficient variations were 6.6% and 12.6%, respectively.

Insulin resistance was measured by the homeostasis model assessment (HOMA). HOMA insulin resistance index (HOMA-IR) was calculated as [Plasma glucose (GLU, mmol/L) × serum insulin (mIU/L)]/22.5. HOMA insulin sensitivity index (HOMA-ISI) was calculated as 1/(GLU × serum insulin).

### Definition of obesity

The age- and sex-specific BMI reference developed by Working Group on Obesity in China (WGOC) was used for defining the overweight and obesity [25]. The 85th and the 95th percentiles were used as cut-off points for defining overweight and obesity, respectively. In present study, overweight included obesity.

### Genotyping

Genomic DNA was isolated from whole blood, using the QIAmp Blood kit (Qiagen). The rs9939609 SNP of the *FTO *gene was selected. This polymorphism was genotyped by allelic discrimination assays using a TaqMan probe allelic discrimination (Apllied Biosystems). Fluorescence was visualized through an ABI 7900 HT fast real-time PCR system (Apllied Biosystems). The genotyping success rate was 99.4%, and genotype distribution was in Hardy-Weinberg equilibrium (*P *= 0.4732). To assess genotyping reproducibility, a random 10% selection of samples was regenotyped with 100% concordance.

### Statistical analysis

A likelihood ratio test was performed to assess Hardy-Weinberg equilibrium. Based on the minor allele frequency of 0.12, a prevalence of 0.25 and a significance level at 0.05, our study was powered at 44% to detect odds ratios (OR) of 1.15 for obesity. Logistic regression analysis was performed to compare the likelihood of overweight and obesity between the carriers of TT genotype and the carrier of AA/AT genotype. Association of *FTO *rs9939609 with obesity-related measures and obesity-related metabolic traits were performed with General Linear Model, using sex, age and puberty stages as covariates. Triglyceride, insulin and leptin were natural logarithm transformed before analysis. When the associations between *FTO *rs9939609 and obesity-related metabolic traits were significant, BMI was further adjusted.

## Results

### Characteristics of subjects

The characteristics of the subjects are given in Table 1. A total of 670 children aged 9.3 ± 0.8 years (332 girls and 338 boys) completed the study.

**Table 1:** Characteristics of the study objects

Characteristics	Total
N	670
Age (years)	9.3 ± 0.8 (8-11)
Girl (%)	49.6
BMI (kg/m^2^)	19.1 ± 3.9
BMI Z-score	0.8 ± 0.7
Overweight and obesity (%)	43.6
Obesity (%)	25.8

Data are means ± SD, or percentages.

The rs9939609 A allele frequency of the study population was 12.2%. The frequency of heterozygote and homozygote of the A allele was 21.9% and 1.2%, respectively. No significant differences in the genotypic distributions were found between boys and girls.

### Association of *FTO *rs9939609 with overweight and obesity

The prevalence of obesity among the carriers of AA/AT genotypes was significantly higher than that among those with TT genotype (36.4% *vs*. 22.6%, *P *= 0.004). Compared to the carrier of TT genotype, the likelihood of obesity was 1.79 (95% confidence interval (95% CI) 1.20-2.67) for the carrier of AA/AT genotype, after adjustment of sex, age and puberty stages, Table 2.

**Table 2:** Associations of the *FTO *variant with overweight and obesity^1^

	Overweight and obesity			Obesity		
		
	Prevalence (%)	OR (95% CI)	*P*	Prevalence (%)	OR (95% CI)	*P*
TT^2^	41.5	1.00		22.6	1.00	
AA/AT	50.7	1.32 (0.91-1.92)	0.15	36.4	1.79 (1.20-2.67)	0.004

^1 ^Logistic regression analyses, adjusted age, sex and puberty stage;

^2 ^Carriers with the TT genotype are used as the reference group.

### *FTO *rs9939609 and obesity-related measures

Compared with the TT genotype, the weight, BMI, BMI Z-score and waist-to-hip ratio (WHR) of carriers with the AA/AT genotype were significantly higher than those with TT genotype, Table 3. Carrier with the AA/AT genotype also had significant higher subscapular skinfold thickness, suprailiac skinfold thickness, body fat and lean body mass as shown in Table 3. The height and percent body fat were not significantly different between the children with TT and AA/AT genotype.

**Table 3:** Association of rs9939609 with obesity-related measures^1^

	TT	AA/AT	*P*
N	515	155	
Weight (kg)	38.1 (0.4)	40.1 (0.8)	0.02
Height (cm)	141.0 (0.3)	141.7 (0.6)	0.30
BMI (kg/m^2^)	18.9 (0.2)	19.8 (0.3)	0.01
BMI Z-score	0.8 (0.1)	1.1 (0.1)	0.02
WHR	0.85 (0.003)	0.86 (0.01)	0.02
Suprailiac skinfold (mm)	18.1 (0.3)	19.5 (0.6)	0.04
Subscapular skinfold (mm)	16.5 (0.5)	19.3 (0.9)	0.003
Fat free mass (kg)	27.5 (0.3)	28.7 (0.5)	0.02
Fat mass (kg)	10.6 (0.2)	11.4 (0.4)	0.049
Percent body fat (%)	27.1 (0.2)	27.5 (0.4)	0.29

Abbreviation: BMI, body mass index; BMI Z-score, body mass index Z-score; WHR, waist to hip ratio.

^1 ^Data are age, sex and puberty stage adjusted mean ± standard error, using General Linear Regression Model.

### *FTO *rs9939609 and obesity-related metabolic traits

The concentration of triglyceride was found obviously higher in the children with AA/AT than carriers with TT genotype (1.13 ± 0.68 mmol/L *vs*. 1.03 ± 0.52 mmol/L, *P *= 0.045). While, the adiponectin levels in subjects with AA/AT genotype were obviously lower than subjects with the TT genotype (16.2 ± 0.7 μg/ml *vs*.18.0 ± 0.4 μg/ml, *P *= 0.03). We also found marginally increasing level of glucose in the AA/AT genotype subjects (4.67 ± 0.40 mmol/L *vs*. 4.60 ± 0.35 mmol/L, *P *= 0.08). The evidence of association was reduced after further adjustment for BMI (*P *= 0.38 for triglyceride, *P *= 0.20 for adiponectin and glucose). There was weak evidence of association between rs9939609 and other obesity-related metabolic traits including TC (3.92 ± 0.03 mmol/L *vs*. 4.02 ± 0.05 mmol/L, *P *= 0.10), insulin (2.69 ± 1.77 ng/ml *vs*. 3.12 ± 2.91 ng/ml, *P *= 0.14), and insulin resistance (HOMA-IR 0.56 ± 0.03 *vs*. 0.66 ± 0.05, *P *= 0.10), Table 4.

**Table 4:** Association of rs9939609 with obesity-related metabolic traits^1^

	TT	AA/AT	*P*	*P *(adjusted)^2^
GLU (mmol/L)	4.60 (0.35)	4.66 (0.40)	0.08	0.20
LogTG	-0.08 (0.02)	0.03 (0.04)	0.045	0.38
TC(mmol/L)	3.92 (0.03)	4.02 (0.05)	0.10	
LDL (mmol/L)	2.24 (0.03)	2.31 (0.05)	0.28	
HDL (mmol/L)	1.41 (0.01)	1.43 (0.02)	0.40	
Loginsulin	0.68 (0.04)	0.80 (0.07)	0.14	
Logleptin	0.57 (0.08)	0.63 (0.15)	0.76	
Adiponectin (μg/ml)	18.0 (0.4)	16.2 (0.7)	0.03	0.20
Ghrelin (ng/ml)	3.72 (0.08)	3.89 (0.15)	0.30	
SBP (mmHg)	104.8 (0.4)	105.9 (0.8)	0.21	
DBP (mmHg)	67.9 (0.3)	67.8 (0.6)	0.91	
HOMA-IR	0.56 (0.03)	0.66 (0.05)	0.10	
HOMA-ISI	0.16 (0.01)	0.14 (0.02)	0.26	

Abbreviation: GLU, fasting glucose; LogTG, natural logarithm-transformed triglyceride; TC, total cholesterol; LDL, low-density lipoprotein-cholesterol; HDL, high-density lipoprotein-cholesterol; Loginsulin, log-transformed insulin; Logleptin, natural logarithm-transformed leptin; SBP, systolic blood pressure; DBP, diastolic blood pressure; HOMA-IR, homeostasis model assessment of insulin resistance index; HOMA-ISI, homeostasis model assessment of insulin sensitivity index.

^1 ^Data are age, sex and puberty stage adjusted mean ± standard error, using General Linear Regression Model.

^2 ^Further adjusted BMI.

## Discussion

In this study, we confirmed the strong association of *FTO *SNP rs9939609 with obesity and BMI among Chinese children, in accord with the recent finding of Xi et al. [18]. The obesity prevalence and BMI of AA/AT genotype carriers were significantly higher than that of their counterparts with the TT genotype. Similar to the results reported by Li H (12%), Chang (12.6%) and the results described in HapMap for Han Chinese population (HCB) [9,15], the rs9939609 A allele frequency was substantially lower in our study as compared with the European populations (12.2 *vs*. 45%), and did not deviate from Hardy-Weinberg equilibrium (*P *= 0.47). Only 1.2% of the Chinese population were homozygote of the A allele, in contrast to 16% in European populations.

In our study, the associations of rs9939609 with obesity were significant among Chinese children. While, among the first study about the association between rs9939609 and BMI among Chinese adults in mainland China, Li H et al. [15] reported no association of *FTO *genetic polymorphism in the intron 1 block (rs9939609, rs8050136, and rs9930506) with obesity in the Chinese adults. Several meta-analysis studies demonstrated significant association of *FTO *and BMI in Asian adults, although their risk allele frequency and effect size were lower compared with Europeans [14,26]. Several other studies also found positive association between *FTO *rs9939609 and the risk of obesity (OR = 1.43, OR = 1.38 and OR = 1.39 respectively) in the East Asian adults [9,12,26]. Recently, Li X et al. [17] found that the A allele was strongly associated with obesity and overweight in Chinese adults (OR = 1.45, 95% CI 1.10-1.90). Meanwhile, Xi et al. [18] firstly confirmed the significant associations of rs9939609 variant with BMI and the risk of obesity in Chinese children and adolescents in Beijing, China. Our study further confirmed the positive association for this variant in Chinese children. Although the effect size estimate of 1.79 seems larger in our study, it does not mean that the effect size is bigger than those reported in Asian adults because the 95% confidence intervals for 1.79 are wide (95% CI 1.20-2.67) and encompass the previously reported estimates. In addition, the effect size of *FTO *rs9939609 on the risk of obesity observed in our study was similar to the effects reported by Frayling et al. in UK children aged 11 years (OR per-A allele = 1.35, 95% CI 1.14-1.61) [3] and Hinney et al. in German children and adolescents (OR = 1.57, 95% CI 1.30-1.90) [27], although the minor allele frequency of rs9939609 SNP was lower in Chinese than in European populations.

The reason for the different results among Chinese populations might be the ages of studied population. Our study recruited children aged 8-11 years; the subjects aged 6-18 years in Xi's study [18] and 45 years in Li X's study [17], all being younger than the study population in Li H's study who were 50-70 years [15]. The approach among young population may increase the genetic load and decrease the interference of environmental effects. Qi et al. reported that the association between rs9939609 and BMI in elderly men decreased [28]. Some studies also suggest that genetic effects influencing obesity may alter throughout life and decrease with older age [29-31]. The interference of environmental effects increases with older age and may exert stronger influences on later BMI. Loos's study also showed that the association between *FTO *and BMI was higher in children than in adults [32]. This discrepancy might be due to inability of BMI to capture adiposity as successfully in older people as in younger people. Aging is characterized by lean body mass loss and adipose tissue increase without weight gain that may not be captured by BMI, and traditional adiposity measures lose their predictive ability in the elderly [33-35].

We also confirmed the finding of Freathy et al. [36] that *FTO *genotype was associated with metabolic traits to an extent consistent with its effect on BMI, which were reduced after adjustment for BMI. Those results implied that the association of *FTO *genotype with plasma triglyceride and adiponectin may be mediated through obesity. In accord with Zabena's report [37], no association between serum leptin levels and rs9939609 genotypes was detected. In according with several studies [10,16,38,39] in Chinese and Japanese populations, there was weak evidence of association between rs9939609 and obesity-related metabolic traits including TC, insulin, and insulin resistance. This may be due to the minor allele frequency in the Chinese Han population and other Asians was much smaller than that in the European population. A larger sample size is needed to achieve sufficient power under a smaller minor allele frequency. Our study only obtained 44% power to detect the association between *FTO *genotype and obesity. It would require more power to detect the associations between *FTO *genotype and other obesity-related metabolic traits.

The limitations of the present study should be noticed. The first is the small sample size and limited statistic power, though it seems to be sufficient to detect the significant association between *FTO *and obesity, it may be not sufficient to detect the association between *FTO *and some obesity-related metabolic traits if it did exist, which need further research. The recruitment procedure may also have some potential impact on the results. As the participation was voluntary, overweight and obese students and/or who pay more attention to health may be more likely to participate. However, we can still infer similar conclusions except that students carrier specific genotype would be more prefer to the study, which was not the case by now. Therefore, we do not consider the selection bias as a major limitation. Moreover, using a single genetic variant of given gene (*FTO *rs9939609) in present study was also a limitation, which may be in linkage disequilibrium with polymorphisms of other nearby genes that actually contributed to the development of obesity.

## Conclusions

In summary, we replicated that the genetic variation in the *FTO *gene associates with obesity in the Chinese children. More replication studies about the association between the *FTO* gene and obesity-related metabolic traits were guaranteed among larger Chinese population.

## Competing interests

The authors declare that they have no competing interests.

## Authors' contributions

HF and YL coordinated the DNA sample collection and genotyping, carried out statistical analyses and drafted the manuscript. SD carried out the field work including sample and data collection. GM, XH, QZ and AL were involved in the project design and helped draft the manuscript. GM supervised the progress of the study and finalized the manuscript critically. All authors read and approved the final manuscript.

## Pre-publication history

The pre-publication history for this paper can be accessed here:

http://www.biomedcentral.com/1471-2350/11/136/prepub

## References

[B1] Willer CJ Speliotes EK Loos RJ Li S Lindgren CM Heid IM Berndt SI Six new loci associated with body mass index highlight a neuronal influence on body weight regulation Nat Genet 2009 41 1 25 34 10.1038/ng.287 19079261 PMC2695662

[B2] Thorleifsson G Walters GB Gudbjartsson DF Steinthorsdottir V Sulem P Helgadottir A Styrkarsdottir U Gretarsdottir S Thorlacius S Jonsdottir I Genome-wide association yields new sequence variants at seven loci that associate with measures of obesity Nat Genet 2009 41 1 18 24 10.1038/ng.274 19079260

[B3] Frayling TM Timpson NJ Weedon MN Zeggini E Freathy RM Lindgren CM Perry JR Elliott KS Lango H Rayner NW A common variant in the *FTO *gene is associated with body mass index and predispones to childhood and adult obesity Science 2007 316 889 894 10.1126/science.1141634 17434869 PMC2646098

[B4] Dina C Meyre D Gallina S Durand E Korner A Jacobson P Carlsson LM Kiess W Vatin V Lecoeur C Variation in *FTO *contributes to childhood obesity and severe adult obesity Nat Genet 2007 39 724 726 10.1038/ng2048 17496892

[B5] López-Bermejo A Petry CJ Díaz M Sebastiani G de Zegher F Dunger DB Ibáñez L The association between the *FTO *gene and fat mass in humans develops by the postnatal age of two weeks J Clin Endocrinol Metab 2008 93 4 1501 1505 10.1210/jc.2007-2343 18252780

[B6] Peeters A Beckers S Verrijken A Roevens P Peeters P Van Gaal L Van Hul W Variants in the *FTO *gene are associated with common obesity in the Belgian population Mol Genet Metab 2008 93 4 481 484 10.1016/j.ymgme.2007.10.011 18055244

[B7] Andreasen CH Stender-Petersen KL Mogensen MS Torekov SS Wegner L Andersen G Nielsen AL Albrechtsen A Borch-Johnsen K Rasmussen SS Low physical activity accentuates the effect of the *FTO *rs9939609 polymorphism on body fat accumulation Diabetes 2008 57 1 95 101 10.2337/db07-0910 17942823

[B8] Scuteri A Sanna S Chen WM Uda M Albai G Strait J Najjar S Nagaraja R Orrú M Usala G Genome-wide association scan shows genetic variants in the *FTO *gene are associated with obesity-related traits PLoS Genet 2007 3 7 e115 10.1371/journal.pgen.0030115 17658951 PMC1934391

[B9] Chang YC Liu PH Lee WJ Chang TJ Jiang YD Li HY Kuo SS Lee KC Chuang LM Common variation in the fat mass and obesity-associated (*FTO*) gene confers risk of obesity and modulates BMI in the Chinese population Diabetes 2008 57 8 2245 2252 10.2337/db08-0377 18487448 PMC2494679

[B10] Tan JT Dorajoo R Seielstad M Sim X Rick OT Seng CK Yin WT Saw SM Kai CS Aung T *FTO *variants are associated with obesity in the Chinese and Malay populations in Singapore Diabetes 2008 57 10 2851 2857 10.2337/db08-0214 18599522 PMC2551698

[B11] Omori S Tanaka Y Takahashi A Hirose H Kashiwagi A Kaku K Kawamori R Nakamura Y Maeda S Association of CDKAL1, IGF2BP2, CDKN2A/B, HHEX, SLC30A8, and KCNJ11 with susceptibility to type 2 diabetes in a Japanese population Diabetes 2008 57 791 795 10.2337/db07-0979 18162508

[B12] Hotta K Nakata Y Matsuo T Kamohara S Kotani K Komatsu R Itoh N Mineo I Wada J Masuzaki H Variations in the *FTO *gene are associated with severe obesity in the Japanese J Hum Genet 2008 53 6 546 553 10.1007/s10038-008-0283-1 18379722 PMC2413114

[B13] Cha SW Choi SM Kim KS Park BL Kim JR Kim JY Shin HD Replication of genetic effects of *FTO *polymorphisms on BMI in a korean population Obesity 2008 16 9 2187 2189 10.1038/oby.2008.314 18551112

[B14] Ng MC Park KS Oh B Tam CH Cho YM Shin HD Lam VK Ma RC So WY Cho YS Implication of genetic variants near TCF7L2, SLC30A8, HHEX, CDKAL1, CDKN2A/B, IGF2BP2, and *FTO *in type 2 diabetes and obesity in 6,719 Asians Diabetes 2008 57 8 2226 2233 10.2337/db07-1583 18469204 PMC2494677

[B15] Li H Wu Y Loos RJ Hu FB Liu Y Wang J Yu Z Lin X Variants in the fat mass- and obesity-associated (*FTO*) gene are not associated with obesity in a Chinese Han population Diabetes 2008 57 1 264 268 10.2337/db07-1130 17959933

[B16] Horikoshi M Hara K Ito C Shojima N Nagai R Ueki K Froguel P Kadowaki T Variations in the HHEX gene are associated with increased risk of type 2 diabetes in the Japanese population Diabetologia 2007 50 12 2461 2466 10.1007/s00125-007-0827-5 17928989

[B17] Li X Song F Jiang H Zhang M Lin J Bao W Yao P Yang X Hao L Liu L A genetic variation in the fat mass- and obesity-associated gene is associated with obesity and newly diagnosed type 2 diabetes in a Chinese population Diabetes Metab Res Rev 2010 26 2 128 132 10.1002/dmrr.1066 20186840

[B18] Xi B Shen Y Zhang M Liu X Zhao X Wu L Cheng H Hou D Lindpaintner K Liu L The common rs9939609 variant of the fat mass and obesity-associated gene is associated with obesity risk in children and adolescents of Beijing, China BMC Med Genet 2010 11 107 10.1186/1471-2350-11-107 20598163 PMC2914647

[B19] World Health Organization Measuring obesity-classification and description of anthropometric data Report on a WHO consultation of the epidemiology of obesity. Warsaw 21-23 October 1987 1989 Copenhagen: WHO

[B20] World Health Organization Computation of centiles and z-scores for height-for-age, weight-for-age and BMI-for-age 2007 WHO http://www.who.int/growthref/computation.pdf (assessed September 2009)

[B21] de Onis M Onyango AW Borghi E Siyam A Nishida C Siekmann J Development of a WHO growth reference for school-aged children and adolescents Bull World Health Organ 2007 85 9 660 667 10.2471/BLT.07.043497 18026621 PMC2636412

[B22] Deurenberg P van der Kooy K Leenen R Weststrate JA Seidell JC Sex and age specific prediction formulas for estimating body composition from bioelectrical impedance: a cross validation study Int J Obes 1991 15 1 17 25 2010255

[B23] Marshall WA Tanner JM Variations in pattern of pubertal changes in girls Arch Dis Child 1969 44 235 291 303 10.1136/adc.44.235.291 5785179 PMC2020314

[B24] Marshall WA Tanner JM Variations in the pattern of pubertal changes in boys Arch Dis Child 1970 45 239 13 23 10.1136/adc.45.239.13 5440182 PMC2020414

[B25] Group of China Obesity Task Force Body mass index reference norm for screening overweight and obesity in Chinese children and adolescents Chin J Epidemiol 2004 25 2 97 102 15132858

[B26] Liu Y Liu Z Song Y Zhou D Zhang D Zhao T Chen Z Yu L Yang Y Feng G Meta-analysis added power to identify variants in *FTO *associated with type 2 diabetes and obesity in the Asian population Obesity (Silver Spring) 2010 18 8 1619 1624 10.1038/oby.2009.469 20057365

[B27] Hinney A Nguyen TT Scherag A Friedel S Brönner G Müller TD Grallert H Illig T Wichmann HE Rief W Genome wide association (GWA) study for early onset extreme obesity supports the role of fat mass and obesity associated gene (FTO) variants PLoS One 2007 2 12 e1361 10.1371/journal.pone.0001361 18159244 PMC2137937

[B28] Qi L Kang K Zhang C van Dam RM Kraft P Hunter D Lee CH Hu FB Fat mass-and obesity-associated (*FTO*) gene variant is associated with obesity: longitudinal analyses in two cohort studies and functional test Diabetes 2008 57 11 3145 3151 10.2337/db08-0006 18647953 PMC2570413

[B29] Fabsitz RR Carmelli D Hewitt JK Evidence for independent genetic influences on obesity in middle age Int J Obes Relat Metab Disord 1992 16 9 657 666 1328090

[B30] Stunkard AJ Foch TT Hrubec Z A twin study of human obesity JAMA 1986 256 1 51 54 10.1001/jama.256.1.51 3712713

[B31] Korkeila M Kaprio J Rissanen A Koskenvuo M Effects of gender and age on the heritability of body mass index Int J Obes 1991 15 10 647 654 1752725

[B32] Loos RJ Lindgren CM Li S Wheeler E Zhao JH Prokopenko I Inouye M Freathy RM Attwood AP Beckmann JS Common variants near MC4R are associated with fat mass, weight and risk of obesity Nat Genet 2008 40 6 768 775 10.1038/ng.140 18454148 PMC2669167

[B33] Janssen I Katzmarzyk PT Ross R Waist circumference and not body mass index explains obesity-related health risk Am J Clin Nutr 2004 79 3 379 384 14985210 10.1093/ajcn/79.3.379

[B34] Stevens J Cai J Thun MJ Williamson DF Wood JL Consequences of the use of different measures of effect to determine the impact of age on the association between obesity and mortality Am J Epidemiol 1999 150 4 399 407 10453816 10.1093/oxfordjournals.aje.a010019

[B35] Visscher TL Seidell JC Molarius A van der Kuip D Hofman A Witteman JC A comparison of body mass index, waist-hip ratio and waist circumference as predictors of all-cause mortality among the elderly: the Rotterdam study Int J Obes Relat Metab Disord 2001 25 11 1730 1735 10.1038/sj.ijo.0801787 11753597

[B36] Freathy RM Timpson NJ Lawlor DA Pouta A Ben-Shlomo Y Ruokonen A Ebrahim S Shields B Zeggini E Weedon MN Common variation in the *FTO *gene alters diabetes-related metabolic traits to the extent expected given its effect on BMI Diabetes 2008 57 5 1419 1426 10.2337/db07-1466 18346983 PMC3073395

[B37] Zabena C González-Sánchez JL Martínez-Larrad MT Torres-García A Alvarez-Fernández-Represa J Corbatón-Anchuelo A Pérez-Barba M Serrano-Ríos M The *FTO *obesity gene. Genotyping and gene expression analysis in morbidly obese patients Obes Surg 2009 19 1 87 95 10.1007/s11695-008-9727-0 18855084

[B38] Horikoshi M Hara K Ito C Shojima N Nagai R Ueki K Froguel P Kadowaki T Variations in the HHEX gene are associated with increased risk of type 2 diabetes in the Japanese population Diabetologia 2007 50 12 2461 2466 10.1007/s00125-007-0827-5 17928989

[B39] Hotta K Nakata Y Matsuo T Kamohara S Kotani K Komatsu R Itoh N Mineo I Wada J Masuzaki H Variations in the *FTO *gene are associated with severe obesity in the Japanese J Hum Genet 2008 53 6 546 553 10.1007/s10038-008-0283-1 18379722 PMC2413114

